# Exploring the Potential of Electronic Patient-Generated Health Data for Evaluating Treatment Response to Intramuscular Steroids in Rheumatoid Arthritis: Case Series

**DOI:** 10.2196/55715

**Published:** 2024-10-28

**Authors:** Mariam Al-Attar, Kesmanee Assawamartbunlue, Julie Gandrup, Sabine N van der Veer, William G Dixon

**Affiliations:** 1 Centre for Epidemiology Versus Arthritis University of Manchester Manchester United Kingdom; 2 Centre for Health Informatics, Division of Informatics, Imaging and Data Science Manchester Academic Health Science Centre University of Manchester Manchester United Kingdom; 3 School of Medical Sciences University of Manchester Manchester United Kingdom; 4 Rheumatology Department Salford Royal Hospital Northern Care Alliance NHS Foundation Trust Salford United Kingdom

**Keywords:** patient-reported outcome measures, remote monitoring, patient-generated health data, mobile health, intramuscular steroid injections, rheumatoid arthritis, app, remote monitoring, case series, symptom tracking, pain score

## Abstract

**Background:**

Mobile health devices are increasingly available, presenting exciting opportunities to remotely collect high-frequency, electronic patient-generated health data (ePGHD). This novel data type may provide detailed insights into disease activity outside usual clinical settings. Assessing treatment responses, which can be hampered by the infrequency of appointments and recall bias, is a promising, novel application of ePGHD. Drugs with short treatment effects, such as intramuscular steroid injections, illustrate the challenge, as patients are unlikely to accurately recall treatment responses at follow-ups, which often occur several months later. Retrospective assessment means that responses may be over- or underestimated. High-frequency ePGHD, such as daily, app-collected, patient-reported symptoms between clinic appointments, may bridge this gap. However, the potential of ePGHD remains untapped due to the absence of established definitions for treatment response using ePGHD or established methodological approaches for analyzing this type of data.

**Objective:**

This study aims to explore the feasibility of evaluating treatment responses to intramuscular steroid therapy in a case series of patients with rheumatoid arthritis tracking daily symptoms using a smartphone app.

**Methods:**

We report a case series of patients who collected ePGHD through the REmote Monitoring Of Rheumatoid Arthritis (REMORA) smartphone app for daily remote symptom tracking. Symptoms were tracked on a 0-10 scale. We described the patients’ longitudinal pain scores before and after intramuscular steroid injections. The baseline pain score was calculated as the mean pain score in the 10 days prior to the injection. This was compared to the pain scores in the days following the injection. “Response” was defined as any improvement from the baseline score on the first day following the injection. The response end time was defined as the first date when the pain score exceeded the pre-steroid baseline.

**Results:**

We included 6 patients who, between them, received 9 steroid injections. Average pre-injection pain scores ranged from 3.3 to 9.3. Using our definitions, 7 injections demonstrated a response. Among the responders, the duration of response ranged from 1 to 54 days (median 9, IQR 7-41), average pain score improvement ranged from 0.1 to 5.3 (median 3.3, IQR 2.2-4.0), and maximum pain score improvement ranged from 0.1 to 7.0 (median 4.3, IQR 1.7 to 6.0).

**Conclusions:**

This case series demonstrates the feasibility of using ePGHD to evaluate treatment response and is an important exploratory step toward developing more robust methodological approaches for analysis of this novel data type. Issues highlighted by our analysis include the importance of accounting for one-off data points, varying response start times, and confounders such as other medications. Future analysis of ePGHD across a larger population is required to address issues highlighted by our analysis and to develop meaningful consensus definitions for treatment response in time-series data.

## Introduction

Remote monitoring using electronic patient-generated health data (ePGHD) is an emerging tool for patients with long-term conditions [1]. These data are collected independently by patients [2], either actively at specified time points (eg, self-reported symptom data) or passively and continuously (eg, sensor data) [3,4]. Smartphone apps and wearable devices can routinely collect ePGHD outside usual clinical environments [4,5].

Frequently and longitudinally collected ePGHD can reveal detailed between-visit insights into patient conditions [6]. They include new types of health data not traditionally collected or evaluated, enriching regular clinical assessments [7] and allowing more accurate identification of patterns over time. Understanding fluctuating disease activity trajectories is key to the care of patients with long-term conditions, but can be hampered in usual clinical settings by infrequent reviews and recall bias [8].

Assessing treatment response is a promising and novel application of ePGHD [9,10]. Traditionally, treatment response is measured at predetermined, infrequent intervals dictated by clinical trial or clinic appointment visits [11], posing challenges in capturing day-to-day symptom patterns. This is particularly problematic for drugs with short treatment effects because patients are unlikely to accurately recall their treatment response at follow-up visits that often take place several months later. Retrospective assessment may over- or underestimate responses [12]; one example is intramuscular steroid therapy in rheumatoid arthritis (RA). Despite its widespread use [13], studies evaluating this therapy’s effectiveness are scarce and heterogeneous in both methodology and results [14-19]. This knowledge gap means that rheumatologists struggle with a common question: “How long will this steroid injection last?”

High-frequency ePGHD, such as daily patient-reported symptoms collected remotely via apps between appointments, promise to bridge this gap. However, there is no established definition for what entails the response to intramuscular steroid therapy using ePGHD. Despite the growing interest in ePGHD [20], the absence of established methods for analyzing and interpreting ePGHD hinders its potential to improve clinical practice and patient outcomes [1]. Rather than drawing conclusions on treatment response, this paper provides a first step toward developing such definitions through an exploratory analysis of ePGHD in 6 patients who received intramuscular steroid injections.

## Methods

### Study Context

The REMORA (REmote Monitoring Of Rheumatoid Arthritis) program is codeveloping and evaluating an app-based symptom-tracking system for RA integrated into the electronic health record [21,22]. Our case series was drawn from a prospective cohort study within the REMORA program that tested a regional infrastructure for collecting and integrating ePGHD as part of usual care pathways. Patients with RA were recruited over 6 months from a rheumatology outpatient clinic in northern England. The included participants were (1) aged >18 years, (2) owned and could use an Android smartphone, and (3) could understand verbal and written English. Research nurses identified and recruited eligible participants at in-person appointments or virtual consultations. People who consented received instructions for downloading the REMORA app and creating an “NHS Login,” a trusted login developed by the National Health Service allowing secure access to multiple digital health services. No additional visits were required beyond regular clinic appointments. Among 74 participants who consented to participate during the 6-month recruitment period in 2021-2022, 32 downloaded the app and contributed at least 1 day of data. Reasons for not downloading or using the app were not explored in the REMORA1.5 study and are not essential for interpreting the case series findings, although nonusers were older and had more active disease [23].

### Case Series Inclusion Criteria

We identified patients who received an intramuscular steroid injection during a 12-month tracking period in 2021-2022 and contributed at least 3 days of symptom data both before and after the injection date.

### Data Items Used for Analysis

ePGHD and clinician-reported data were collected for the prospective REMORA cohort study. These included intramuscular steroid use (date of administration, type, dose) as the exposure, and self-reported pain (on a 0-10 scale, with 10 being the worst pain) as the outcome. Other data included demographics (age, sex, ethnicity); date of RA diagnosis; an RA-specific disease activity measure, the Disease Activity Score-28 (DAS-28; score range 0-9.4) at recruitment; and start/stop dates for concomitant disease-modifying antirheumatic drugs (DMARDs). Data on use of over-the-counter analgesics were not collected.

### Treatment Response Follow-Up Period

Pain scores tracked for up to 10 days preceding and up to 8 weeks following the intramuscular injection were reviewed. The data completion rate was calculated as the number of days on which patients reported symptoms during the 8-week postinjection follow-up period.

### Definition of Treatment Response Using ePGHD

Treatment response was defined based on longitudinal symptom data, initially by identifying key response variables and subsequently using these to calculate response definitions (Table 1).

**Table 1:** Key response variables and definitions.

Key response variables	Definitions
Pre-injection pain score	Mean pain score using all pain scores available in the 10 days preceding injection
Response start time	For responders; the day following injection
Response end time	For responders; first date following injection with pain score greater than pre-injection pain score
Response	Pain score lower than pre-injection pain score on the first day following injection (yes/no)
Response duration	Number of days between response start time and response end time
Average pain score during response	Mean pain score during response duration
Nadir pain score during response	Lowest pain score during response duration
Average pain score improvement	Difference between pre-injection pain score and average pain score during response
Maximum pain score improvement	Difference between pre-injection pain score and nadir pain score response

### Ethical Considerations

REMORA1.5 received ethical approval from the UK Health Research Authority and Health and Care Research Wales (21/NW/0007). All participants provided informed written or verbal consent for study participation and use of their deidentified data for future studies. They received no compensation.

## Results

Our case series included 6 patients who received ≥1 intramuscular steroid injection with a total symptom-tracking period of 166-301 days during the REMORA study. The steroid-response tracking period was 42-56 days postinjection, with a data-completion range of 41%-79% of possible days during the 8-week follow-up period; most participants contributed symptom data on at least 50% of days.

Participants included 4 men and 2 women (age range 30-65 years); 5 of 6 were White British. At study recruitment, disease duration varied from newly diagnosed (<1 year) to long-standing (≥12 years); DAS-28 score range was 3.4-7.2. Concomitant DMARDs used during the study period included methotrexate, sulfasalazine, and hydroxychloroquine. Three patients changed DMARDs during the tracking period.

In total, 9 injections were given in the study period, with 1 participant receiving 3 injections over 181 days; we considered each injection a separate case in our series. Steroids included triamcinolone or methylprednisolone; doses ranged from 120 to 180 mg.

**Figure 1:**
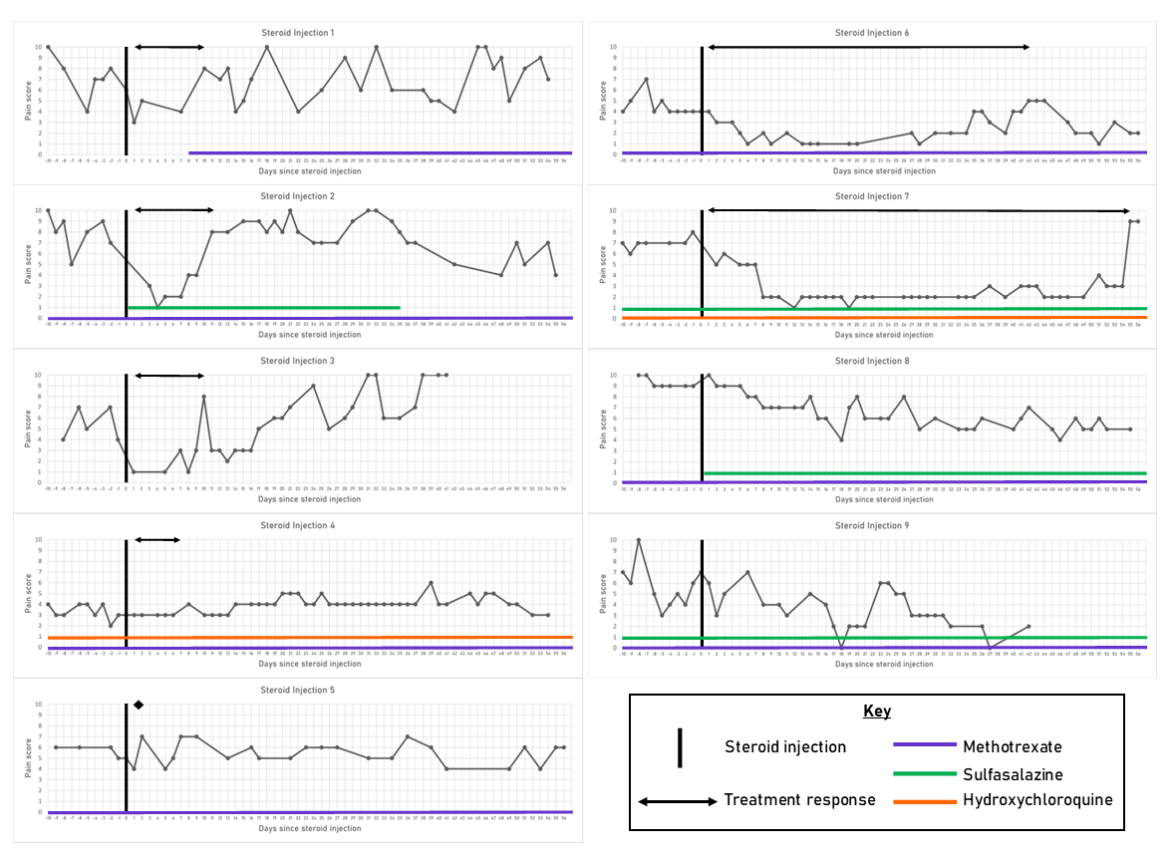
Treatment response patterns for individual steroid injections showing trends in pain score over time. These graphs demonstrate daily pain score fluctuations prior to and following intramuscular steroid injections. Using our definitions, steroid injections 1-7 show a response, whereas injections 8-9 do not. The vertical lines demonstrate the date of steroid injection, and the horizontal arrows represent the treatment response period. Colored horizontal lines represent concomitant disease-modifying antirheumatic drugs received during the study period.

Table 2 summarizes our results. Average pre-injection pain-score range was 3.3-9.3. Using our definitions, 7 injections demonstrated a response and 2 did not. Among responders, response duration range was 1-54 (median 9, IQR 7-41) days; average pain score improved 0.1-5.3 (median 3.3, IQR 1.7-4.4) points; maximum pain score improved 0.1-7.0 (median 4.3, IQR 1.7 to 6.0) points. Among nonresponders (injections 8 and 9), maximum pain score improvement was a negative value indicating the degree of worsening between the pre-injection and day 1 pain score; these negative values have been reflected in the overall median/IQR results reported for maximum pain score improvement in Table 2 (median 3.6, IQR -0.15-5.2). Figure 1 graphically demonstrates patient-level treatment response trajectories for each steroid injection.

**Table 2:** Individual treatment response and demographic data for each steroid injection.

Injection number (patient number)	Data completion rate (%)	Concomitant drugs during analysis period	Intramuscular steroid dose (mg)	Response? (Y/N)	Response duration (days; range 1-54; median 9, IQR 7-41)	Pre-injection pain score (range 3.3-9.3; median 5.8, IQR 5-7.65)	Average pain score response (range 1.8-4.0; median 2.7, IQR 2.2-4.0)	Nadir pain score response (range 1.0-4.0; median 1.0, IQR 1-3)	Average pain score improvement (range 0.1-5.3; median 3.3, IQR 1.7-4.4)	Maximum pain score improvement (range -0.7-7.0; median 3.6, IQR –0.15 to 5.2)
1 (1)	50	MTX^a^	120	Y	9	7.3	4.0	3.0	3.3	4.3
2 (1)	55	MTX, SSZ^b^	120	Y	10	8.0	2.7	1.0	5.3	7.0
3 (1)	50	None	180	Y	9	5.4	1.8	1.0	3.6	4.4
4 (2)	79	MTX, HCQ^c^	120	Y	7	3.3	3.0	3.0	0.1	0.1
5 (3)	41	MTX	160	Y	1	5.8	4.0	4.0	1.7	1.7
6 (4)	61	MTX	120	Y	41	4.6	2.2	1.0	2.4	3.6
7 (5)	77	SSZ, HCQ	120	Y	54	7.0	2.6	1.0	4.4	6.0
8 (3)	68	MTX, SSZ	160	N	—^d^	9.3	—	—	—	–0.7
9 (6)	48	MTX, SSZ	120	N	—	5.6	—	—	—	–0.4

^a^MTX: methotrexate.

^b^SSZ: sulfasalazine.

^c^HCQ: hydroxychloroquine.

^d^Not applicable.

## Discussion

Our study describes a case series of patients who used a smartphone app to collect high-frequency ePGHD, providing a novel opportunity for detailed insights into treatment response. We created and applied definitions to determine the presence, duration, and extent of treatment response to intramuscular steroid administration. Using these definitions, 7 of 9 injections demonstrated a response, with variable extent and duration. We also present graphical representations of ePGHD, which is an innovative way of communicating and understanding the treatment response. Interestingly, the graphical patterns were inconsistent with established beliefs among rheumatologists. Steroid injections 6, 7, and 8 most closely represented expected treatment-response trajectories (ie, rapid, sustained pain-score improvement over months); however, injection 8 did not meet our definition for treatment response, as the day 1 pain score was higher than baseline. Thus, treatment response definitions must be developed to account for within-person variation in symptom scores, response start times, and overall trajectories, as would be usual in clinical practice.

The study population was limited, as this was a secondary use of data from REMORA, which was not designed to evaluate treatment responses. Furthermore, our definitions were designed as a proof of concept for this novel approach in a small dataset and are admittedly arbitrary. Nonetheless, this study is an important exploratory first step toward a robust methodology to evaluate the treatment response using ePGHD. The highlighted issues require addressing before this method is applied to larger datasets and meaningful definitions are developed. For instance, we used pain score data because pain is a common indication for steroid injections; but patients may feasibly demonstrate responses in other domains. Indeed, our cohort’s variation in baseline pain score points to other indications for steroid injection. The most relevant domains need to be identified by exploring the data and collaborating with stakeholders. Furthermore, symptom scores are inherently subjective and can have considerable interuser variability, hindering comparison of scores between individuals. Definitions must be developed that acknowledge within-person changes and account for confounding factors such as comorbidities and concomitant medications. New treatment response definitions also need validation against existing clinical-response measures [24], acknowledging that the infrequency of clinical assessments limits existing measures. Finally, potential barriers to mobile health interventions, such as the burden of daily symptom tracking and health equity considerations that can influence access to smartphones, need to be explored and mitigated [25].

In conclusion, our analysis demonstrates that ePGHD may facilitate novel ways of evaluating treatment responses, potentially filling a long-standing knowledge gap regarding treatment responses to intramuscular steroids and treatment trajectories after any intervention. Further development of ePGHD analysis methodologies across larger populations is required to develop meaningful consensus definitions for treatment responses using longitudinal, patient-reported symptom data.

### Data Availability

The datasets generated during and/or analyzed during this study are available from the corresponding author on reasonable request.

## Abbreviations

DAS-28: Disease Activity Score-28
DMARD: disease-modifying antirheumatic drug
ePGHD: electronic patient-generated health data
RA: rheumatoid arthritis
REMORA: REmote Monitoring Of Rheumatoid Arthritis
